# Synergistic information in a dynamical model implemented on the human structural connectome reveals spatially distinct associations with age

**DOI:** 10.1162/netn_a_00146

**Published:** 2020-09-01

**Authors:** Davide Nuzzi, Mario Pellicoro, Leonardo Angelini, Daniele Marinazzo, Sebastiano Stramaglia

**Affiliations:** Dipartimento Interateneo di Fisica, Universitá degli Studi Aldo Moro, Bari and INFN, Bari, Italy; Dipartimento Interateneo di Fisica, Universitá degli Studi Aldo Moro, Bari and INFN, Bari, Italy; Dipartimento Interateneo di Fisica, Universitá degli Studi Aldo Moro, Bari and INFN, Bari, Italy; Department of Data Analysis, Ghent University, Ghent, Belgium; Dipartimento Interateneo di Fisica, Universitá degli Studi Aldo Moro, Bari and INFN, Bari, Italy; Center of Innovative Technologies for Signal Detection and Processing (TIRES), Universitá degli Studi Aldo Moro, Bari, Italy

**Keywords:** Information theory, Aging, Ising model

## Abstract

We implement the dynamical Ising model on the large-scale architecture of white matter connections of healthy subjects in the age range 4–85 years, and analyze the dynamics in terms of the synergy, a quantity measuring the extent to which the joint state of pairs of variables is projected onto the dynamics of a target one. We find that the amount of synergy in explaining the dynamics of the hubs of the structural connectivity (in terms of degree strength) peaks before the critical temperature, and can thus be considered as a precursor of a critical transition. Conversely, the greatest amount of synergy goes into explaining the dynamics of more central nodes. We also find that the aging of structural connectivity is associated with significant changes in the simulated dynamics: There are brain regions whose synergy decreases with age, in particular the frontal pole, the subcallosal area, and the supplementary motor area; these areas could then be more likely to show a decline in terms of the capability to perform higher order computation (if structural connectivity was the sole variable). On the other hand, several regions in the temporal cortex show a positive correlation with age in the first 30 years of life, that is, during brain maturation.

## Author Summary

In a previous study implementing the Ising model on a 2D lattice, we showed that the joint synergistic information shared by two variables on a target one peaks before the transition to an ordered state (critical point).

Here we implemented the same model on individual structural connectomes, to answer these questions:

-  Does the synergy still peak before the critical point in a nonuniform network?
-  Are the hubs of structural connectivity also hubs of synergy?
-  Is there association with age?

We found that synergy still peaks before the critical temperature and that hubs of structural connectivity are not among the nodes towards which synergy is highest. Furthermore, using robust measures of association we found both positive and negative associations of synergy with age, in localized clusters. In some regions this association is continuous with age; in others it is limited to around the first 30 years of age.

## INTRODUCTION

Recent advances in diffusion imaging and tractography methods allow noninvasive in vivo mapping of white matter cortico-cortical projections at relatively high spatial resolution, thus providing a connection matrix of interregional structural connectivity (SC) representing the *geometry* of the brain (Sporns, 2010). Dynamical models implemented on the large-scale architecture of the human brain may shed light on how function is constrained by the underlying structure. This is the case of the so-called neural mass or mean-field models that describe the collective activity of cell populations (Deco & Jirsa, 2012), or phase coupling models (Finger et al., 2016), down to abstract models such as the Ising model (Deco, Senden, & Jirsa, 2012; Haimovici, Tagliazucchi, Balenzuela, & Chialvo, 2013; Marinazzo et al., 2014; Stramaglia et al., 2017). In particular, some studies showed that the resting activity exhibits peculiar scaling properties, resembling the dynamics near the critical point of a second-order phase transition (Chialvo, 2010). Noise-induced synchronization on the human connectome has been recently studied in Pang, Gollo, and Roberts (2020). Moreover, the possible origin and role of criticality in living adaptive and evolutionary systems has recently been ascribed to adaptive and evolutionary functional advantages (Hidalgo et al., 2014).

In this paper we implement a dynamical model on the individual large-scale structural connectivity of healthy subjects in the age range 4–85 years, and look for the dynamical properties of the simulated dynamics that are associated with aging.

Human aging is the set of characteristics that change over time, signifying someone as older or younger. These changes occur at different hierarchical levels, called metrics of aging: biological aging, phenotypic aging, and functional aging (Ferrucci, Levine, Kuo, & Simonsick, 2018). Connectomics (Bassett & Sporns, 2017) provides a unique resource for examining how brain organization and connectivity changes across typical aging, both in terms of plasticity and function, and in terms of how these differences relate to brain disorders. Several studies have used diffusion imaging to investigate changes in structural connectivity over the healthy human life span. In general, these studies have observed a nonlinear inverted U-shaped trajectory association between age and fractional anisotropy (FA), and a U-shaped trajectory (opposite to FA) for axial, mean, and radial diffusivity (Westlye et al., 2010). A review of age-related disruption of the brain’s regional integrity and information flow across the brain, as expressed by age-related differences in functional and structural connectivity, can be found in Damoiseaux (2017).

Considering the structural connectivity network provided by diffusion imaging as a complex network, some global metrics of the networks have been found to be correlated with age (Wozniak & Lim, 2006). Age showed significant positive correlation to the integrated cost but significant negative correlation to the integrated local efficiency, while it showed no significant correlation to the integrated global efficiency (Sun, Tong, & Yang, 2012). The reorganization with age of the whole-brain structural and functional connectivity has been described in Betzel et al. (2014). The age-related alterations in the topological architecture of the white matter structural connectome has been also studied in Zhao et al. (2015), where it has been found that hub integration decreased linearly with age, with loss of frontal hubs and their connections, and that age-related changes in structural connections were predominantly located within and between the prefrontal and temporal modules. Particularly interesting is also the study of the cognitive performance in healthy older adults, which in Cabral, Vidaurre, and Marques (2017) has been related to the switching behavior of resting functional connectivity, and in Hinault, Larcher, Bherer, Courtney, and Dagher (2019) to the preservation of structural and effective connectivity. The genetic basis of neurocognitive decline and reduced white matter integrity in normal human brain aging has been studied in Glahn et al. (2013), demonstrating a heritable component for neurocognitive deterioration with age. Concerning brain maturation, positive correlation between structural and functional connectivity has been described in Hagmann et al. (2010), where in addition it has been observed that this relationship strengthened with age. A study of structural brain network changes across the adult life span reported 16 significant age-related networks, with weights decreasing with age linearly in all networks except from the temporal lobe-related network where the decrease was quadratical (Liu et al., 2017). A recent study (Bonifazi et al., 2018) explored joint functional-structural network characteristics that are significantly correlated with aging.

In order to find how a changing structural architecture might influence information processing of a system whose dynamics are constrained by it, in this paper we implement the Ising model on the structural geometry of the brain, here estimated from diffusion tensor imaging. The dynamical properties of the simulated activity that we will consider are derived from the formalism of information decomposition of target effects from multisource interactions, that is the definition of redundant and synergistic components of the information that a set of source variables provide about a target (Lizier, 2012). Applying this framework to the two-dimensional Ising model as a paradigm of a critically transitioning system, and disentangling the components of the information both at the static level and at the dynamical one, it has been recently shown that a key signature of an impending phase transition (approached from the disordered side) is the evidence that the synergy peaks in the disordered phase, both considering only instantaneous interactions, and also considering lagged ones: The synergy can thus be considered a precursor of the transition (Marinazzo, Angelini, Pellicoro, & Stramaglia, 2019).

This study aims to answer three main questions:

•  Does the synergy still peak before the critical point in the nonuniform structural connectvity network?
•  Are the hubs of structural connectivity also hubs of incoming synergy?
•  How does an aging connectome modulate these patterns?

## MATERIALS AND METHODS

### Data

In this work we analyze data form the NKI-Rockland life span study (Nooner et al., 2012), in particular the already processed connectome data provided by the USC Multimodal Connectivity Database (Brown, Rudie, Bandrowski, Van Horn, & Bookheimer, 2012), consisting of 196 connectomes of healthy subjects based on 3T dMRI acquisition (voxel size, 2 mm^3^; 64 gradient directions; TR, 10,000 ms; TE, 91 ms; further details are provided in Brown et al., 2012). The resulting structural connectivity matrices *J*_*ij*_ consist of *N* = 188 regions of interest (ROIs), obtained using the Craddock atlas (Craddock, James, Holtzheimer, Hu, & Mayberg, 2012), linked together by weighted connections based on the number of streamlines connecting pairs of ROIs. The matrix *J*_*ij*_ is symmetric by construction, thus giving rise to a weighted undirected graph. The age of subjects ranges from 4 to 85 years. We consider the individual structural connectivity matrices, and the average connectivity matrix $J_{ij}^{\text{avg}}$ obtained averaging over all subjects.

### Theoretical Framework and Implementation

First we implement the Ising model, with Glauber dynamics, on the average connectome network $J_{ij}^{\text{avg}}$, using the following Hamiltonian:

$$𝓗=-\frac{1}{2}\overset{N}{\underset{i,j=1}{\sum }}J_{ij}s_{i}s_{j},$$ (1)

and updating rules given by

$$p(s_{i}\to -s_{i})=\frac{1}{1+\exp\left(\beta \Delta E_{i}\right)},$$ (2)

where Δ*E*_*i*_ = 2*s*_*i*_ ∑_*j*_ *J*_*ij*_*s*_*j*_ represents the variation of the total energy of the system following the flip of the spin *s*_*i*_. Varying the inverse temperature *β*, the susceptibility shows a peak that identifies the *critical state* of this finite-size system, related to a phase transition occurring in the limit of large networks (Dorogovtsev, Goltsev, & Mendes, 2008). It is worth mentioning that recently this stretching of criticality (from a single point to a more relaxed regime) observed in dynamical models defined on brain networks has been described in the frame of Griffith’s phases (Moretti & Muñoz, 2013). Simulations are initially run for a relaxation time of 10^5^ updates for the first value of *β*, then we start the following procedure: We vary the temperature adiabatically, discard the first 10^4^ updates, and then collect the following 10^6^ updates for statistics. The procedure is repeated for 20 runs at each of the 80 temperature points enclosing the phase transition. We make sure that the state of each spin is updated exactly one time for each iteration of the Glauber dynamics; this allows us to collect one time series *s*_*i*_(*t*) for every spin, where *t* is a discrete time index running from 0 to the total simulation time. In order to characterize the flow of information between the time series in the context of information theory, we interpret each *s*_*i*_(*t*) as a single realization of a discrete-time stationary stochastic process *S*_*i*_(*t*), but for the sake of simplicity we will use the notation *s*_*i*_(*t*) also to refer to the stochastic process. Joint probabilities used in the calculations of information quantities are obtained directly from the data samples as the frequency of each configuration.

The flow of information between variables can be measured in the framework of information dynamics using the transfer entropy (TE), a quantity introduced in Schreiber (2000) and based on appropriate conditioning of transition probabilities (Bossomaier, Barnett, Harré, & Lizier, 2016). Unlike mutual information *I*(*X*; *Y*), a symmetric quantity that measures only the information that is statistically shared by the variables *X* and *Y*, transfer entropy is an inherently asymmetrical quantity and can effectively distinguish between driving and responding elements. Let *s*_*i*_ be the stochastic process associated with a given spin, taken as the target variable, and let ${\tilde{s}}_{i}$ be the same process one time step in the future. Let {*s*_*j*_, *s*_*k*_, …, *s*_*r*_} be a group of spins that are assumed to be drivers for the spin *s*_*i*_. The transfer entropy from the group of drivers to the target is defined as

$${𝒯}_{\{jk\ldots r\}\to i}=I\left({\tilde{s}}_{i};\{s_{j},\ldots ,s_{r}\}|s_{i}\right).$$ (3)

The choice of different groups of source variables leads to the definition of various information quantities that are commonly found in the literature. If we choose only one source variable *s*_*j*_ and we average over all possible sources and targets, we get the pairwise transfer entropy; it has been shown that this measure peaks at the critical point both for the Ising model on a regular 2D lattice and on brain graphs (Barnett, Lizier, Harré, Seth, & Bossomaier, 2013; Marinazzo et al., 2014). If we choose all the spins but *s*_*i*_ as sources and then average over all possible targets, we get the global transfer entropy (GTE). Information flow, as quantified by GTE, peaks in the paramagnetic phase and is thus able to predict an imminent transition (Barnett et al., 2013). Unfortunately numerical estimation of this quantity is unfeasible in systems involving a large number of drivers for each target. As discussed in J. T. Lizier, Prokopenko, and Zomaya (2010) GTE is a measure of collective information transfer, capturing both pairwise and higher order (multivariate) correlations to a variable. It follows that explicitly disentangling the components of the collective information flow is needed to get a better description of the system in the proximity of the transition. As it has been demonstrated in Marinazzo et al. (2019) using the information decomposition frame first introduced in Williams and Beer (2010) and Williams and Beer (2011), considering as few as two sources at the same time is sufficient to construct a precursor of the transition. Let *s*_*i*_ be the target spin and *s*_*j*_, *s*_*k*_ be two different driver spins; then the desirable information decomposition is

$${𝒯}_{jk\to i}={𝒰}_{j\to i}+{𝒰}_{k\to i}+{𝓡}_{jk\to i}+{𝒮}_{jk\to i},$$ (4)

$${𝒯}_{j\to i}={𝒰}_{j\to i}+{𝓡}_{jk\to i},$$ (5)

$${𝒯}_{k\to i}={𝒰}_{k\to i}+{𝓡}_{jk\to i}.$$ (6)

The terms 𝒰_*j*→*i*_ and 𝒰_*k*→*i*_ quantify the components of the information about the future of spin *s*_*i*_ that are unique to the sources *s*_*j*_ and *s*_*k*_, respectively. The term 𝓡_*jk*→*i*_ describes the redundant information, the component that is shared between the two source variables. Finally, the term 𝒮_*jk*→*i*_ quantifies the synergy between the sources, intended as the amount of information that can be acquired only considering the sources together, but not considering them alone. Synergy is the only term in this decomposition that contains higher order correlations that can’t be captured by pairwise measures (J. Lizier, Bertschinger, Jost, & Wibral, 2018).

Shannon theory of information does not include a definition for synergy and redundancy; in fact, there are many different conceptual definitions for those two quantities (Griffith, Chong, James, Ellison, & Crutchfield, 2014; Harder, Salge, & Polani, 2013; Quax, Har-Shemesh, & Sloot, 2017). The information decomposition provided in Equation (4) contains only three equations for the four unknown quantities 𝒰_*j*→*i*_, 𝒰_*k*→*i*_, 𝓡_*jk*→*i*_, 𝒮_*jk*→*i*_, therefore the definition of only one of them is needed in order to solve the system. The so-called minimum mutual information (MMI) PID (Barrett, 2015) gives a definition for the redundancy: It assumes that the information that is shared between the sources, independently of the correlations between them, can be identified with the minimum of the information provided by each individual source to the target. Specifically

$${𝓡}_{jk\to i}=\min\left\{{𝒯}_{j\to i},{𝒯}_{k\to i}\right\}.$$ (7)

Another choice for the information decomposition is given in Bertschinger, Rauh, Olbrich, Jost, and Ay (2014) and is based on the following idea. Let *p*(*s*_*i*_, *s*_*j*_, *s*_*k*_) be the joint probability distribution of the three spins and let *p*(*s*_*i*_, *s*_*j*_), *p*(*s*_*i*_, *s*_*k*_) be the two marginal distributions that involve the target and only one driver. Then the redundancy and the unique information should depend on only the marginal distributions but not the particular choice of *p*(*s*_*i*_, *s*_*j*_, *s*_*k*_), which should only affect the synergy. If we define Δ_*p*_ as the set of all the trivariate probability distributions *q* that gives rise to the same marginal distributions as *p*, that is, *q*(*s*_*i*_, *s*_*j*_) = *p*(*s*_*i*_, *s*_*j*_), *q*(*s*_*i*_, *s*_*k*_) = *p*(*s*_*i*_, *s*_*k*_), we can define the synergy as

$${𝒮}_{jk\to i}={𝒯}_{jk\to i}-\underset{q\in \Delta_{p}}{\min}{\tilde{𝒯}}_{jk\to i},$$ (8)

where $\tilde{𝒯}$ is the transfer entropy evaluated using *q* as the joint probability distribution. It has been shown (Barrett, 2015) that in the case of Gaussian stochastic variables those two approaches are equivalent and provide the same decomposition. We have verified empirically that the same holds true for the problem at hand.

The average connectome is a dense network, hence for the dynamics on the average connectome we take into account all the triplets of brain nodes and evaluate the information decomposition for each triplet: In order to evaluate the typical incoming synergy for each node, 𝒮 is averaged over all the triplets having that node as a target.

The individual structural networks, on the other hand, have many nearly zero entries; it is a sparse network: Therefore we make a selection of relevant triplets of brain nodes {*j*, *k*, *i*}, with target *i*, requiring that both *J*_*ji*_ and *J*_*ki*_ are higher than a threshold *J*_*th*_. We fix *J*_*th*_ so as the total number of considered links is 20% of all the possible pairs, and verify that our results are robust w.r.t. the choice of *J*_*th*_. For all the selected triplets we evaluate the information decomposition and, in order to evaluate the typical incoming synergy for each node of an individual network, we average the synergy over all the triplets, among the selected ones, that have that node as the target.

## RESULTS

### Ising Model on the Average Connectivity Network

**Figure 1.**
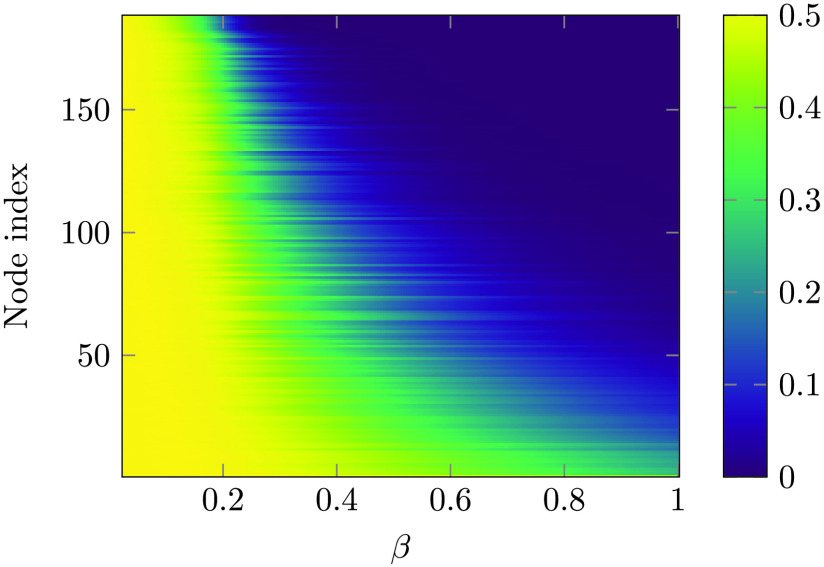
The probability of flip, for each spin at one time step, is depicted as a function of *β*. The nodes are ordered according to increasing strength of the average connectome. At low temperatures spins *freeze*, each spin freezing at a different temperature; the figure shows that the strength of nodes explains, to a certain extent, the variability of the freezing temperature.

Because of the range of strengths of each node, each spin undergoes a dynamical transition at a different temperature. This phenomenon can be visualized studying the probability for the spin to flip at each time step, shown in Figure 1. For *β* = 0 every spin has exactly a 50% chance to flip, while as *β* gets larger the probability drops to 0 at a different rate, depending on the strength (for each node, the strength is the sum of the weights of its connections) of the corresponding node; ordering the nodes by their strength shows that stronger nodes tend to freeze at higher temperatures. As already noted in Marinazzo et al. (2014), as the temperature is lowered the subnetwork constituted by the hubs (we verified the average connectivity network is a rich club) tend to align and to build a cluster of highly correlated spins with slow dynamics. More and more spins are recruited to join this cluster as the temperature is further lowered, until all the system is magnetized. As a consequence, this system does not show a critical temperature, rather it is characterized by a critical range of temperatures, in accordance with Griffith’s theory (Moretti & Muñoz, 2013).

**Figure 2.**
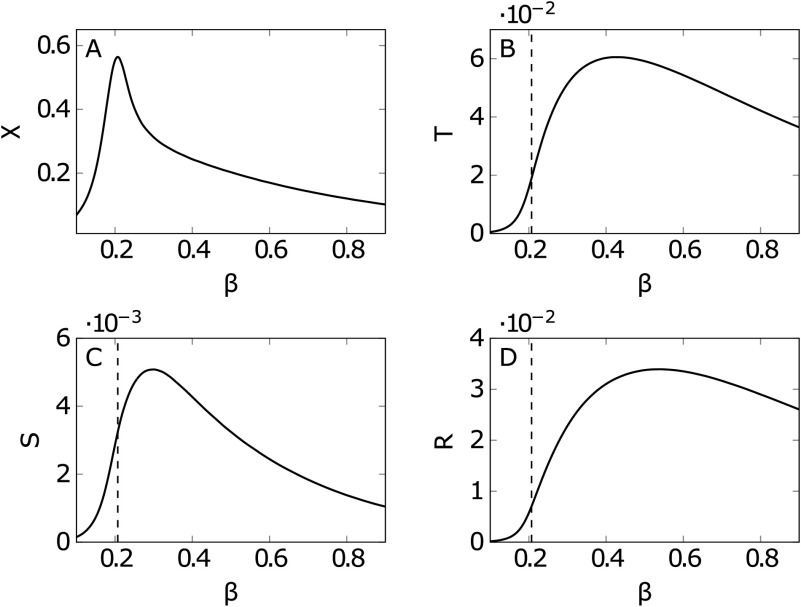
Information decomposition for the Ising model on the brain connectome. (A) Susceptibility *χ* versus the inverse temperature *β*; the temperature where it peaks is considered as the critical state of the system. (B)(C)(D) Trivariate transfer entropy, redundancy, and synergy averaged over all spin triplets, depicted against *β*, peaking in the ferromagnetic state. The dashed line indicates the critical temperature.

**Figure 3.**
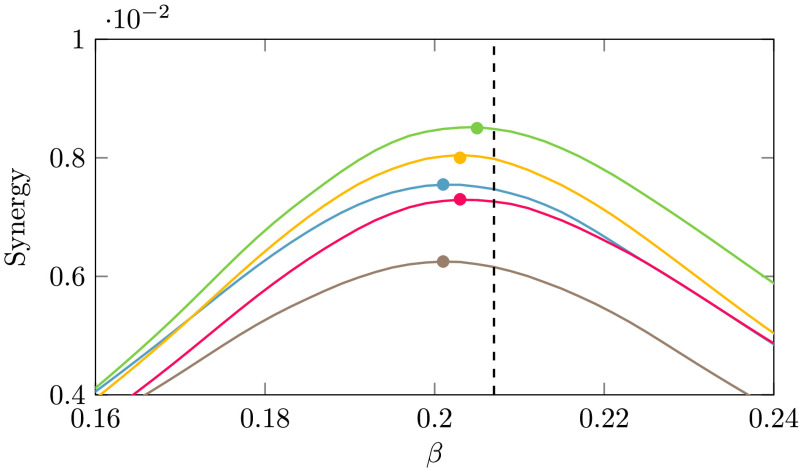
The average synergy towards the five brain nodes with highest strength is depicted versus *β*; their plot peaks before the critical temperature, here identified as the peak of susceptibility.

**Figure 4.**
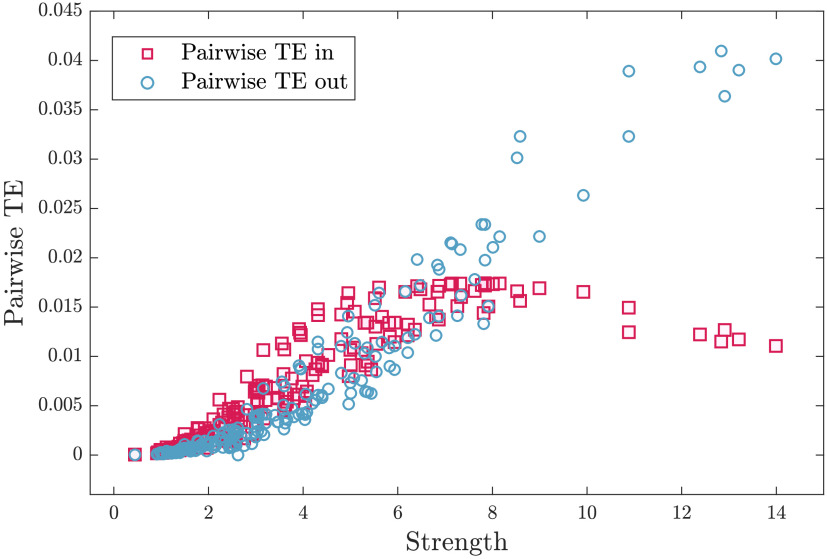
Incoming and outgoing transfer entropy. The amount of information that a fixed driver node transfers to all possible targets (typical outgoing TE) increases steadily with the strength of the node. On the other hand, the amount of information received by a target node from all the drivers (typical incoming TE) behaves similarly for small strength values but decreases above a certain strength threshold.

In Figure 2 we have depicted, as a function of the coupling *β*, the global quantities related to the information decomposition, that is, the trivariate transfer entropy, the average synergy, and the redundancy, as well as the susceptibility *χ*, the classical index of criticality. Looking at the figure, we see that the peak of *χ* precedes, as *β* increases, the peaks of the synergy, the transfer entropy, and the redundancy in order. The peak of *χ* in this heterogeneous system occurs at lower *β* than the peak of the synergy, different from what happens in the Ising model on a regular lattice (Marinazzo et al., 2019); however, one may consider the synergy for each target node, averaged over the pairs of driving variables, and realize that each node experiences its maximum of the synergy at different temperatures. As depicted in Figure 3, the hubs show their peak of synergy before the peak of *χ*, hence their synergy can be seen as precursor of the transition to order. A weighted average of the synergy of each node, with properly fixed weights concentrated on the hubs of the strength, would also peak before criticality and thus would constitute a global precursor of the transition. Another interesting remark to be made is that, as noticed for example in Novelli, Atay, Jost, and Lizier (2019), the pairwise transfer entropy from a source to a target node in a network does not depend solely on the local source-target link weight, but on the wider network structure that the link is embedded in. Deeply connected is the fact that the information flow in networks obeys the law of diminishing marginal returns; see Marinazzo, Wu, Pellicoro, Angelini, and Stramaglia (2012). In Figure 4 we depict the incoming and outgoing transfer entropy of each node, as a function of the strength of the node, where the temperature is fixed at the peak of *χ*. Because of the limited capacity of a spin to encode the incoming information, nodes with high strength send more information to the system than it gets; nodes with low strength send and receive approximately the same amount of information. These findings support the picture of a dynamics shaped by the hubs of structural connectivity.

**Figure 5.**
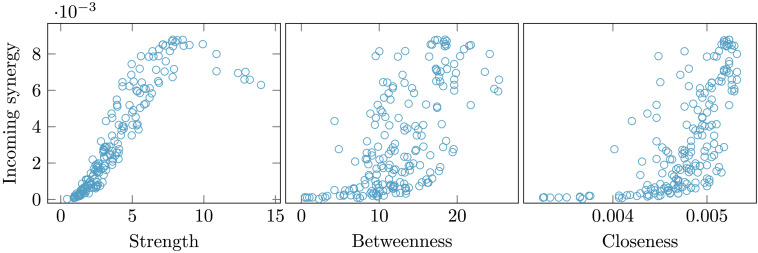
Comparing synergy with topological indices. From the left to the right, for each brain node the typical incoming synergy at criticality is compared with the strength of nodes, the betweenness, and the closeness.

Here we show that a similar behavior holds for the average incoming synergy to each given node, in other words when we average the synergy of all the triplets with the given node as the target. In Figure 5 the incoming synergy, at criticality, is depicted versus the topological character of nodes: strength, betweenness and closeness. We see that the hubs of structural connectivity, which as described above are the drivers of the dynamics, are not among the nodes towards which synergy is highest. Measures of centrality, like betweenness and closeness, appear to be more associated with synergy than strength: The joint state of brain regions is more likely to be projected in *central* regions of the brain.

**Figure 6.**
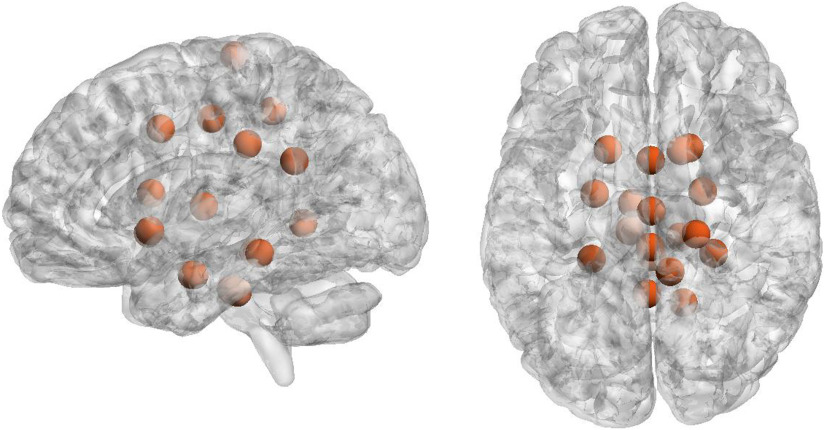
Hubs of synergy. Top nodes for the value of incoming synergy, radius and color of the spheres are arbitrary: right hippocampus, brain stem, right parahippocampal posterior left Parahippocampal posterior, right Cingulate posterior, right precentral, left thalamus, left parahippocampal posterior, left hippocampus, right lingual, right caudate, right cingulate anterior.

In Figure 6 we have depicted the 10% nodes with highest average incoming synergy. Notably these nodes correspond to hippocampus and parahippocampal regions, which are supposed to play important roles in higher order cognitive functions, specifically learning and memory processes (Nemanic, Alvarado, & Bachevalier, 2004); the brain stem, which controls the flow of messages between the brain and the rest of the body, as well as several body functions; the cingulate cortex, which has been associated with several complex cognitive functions, such as empathy, impulse control, emotion, and decision-making; and the thalamus, whose main function is to relay motor and sensory signals to the cerebral cortex.

These two figures were plotted with BrainNet Viewer (Xia, Wang, & He, 2013).

### Ising Model on Individual Connectivity Networks and Correlations with Aging

**Figure 7.**
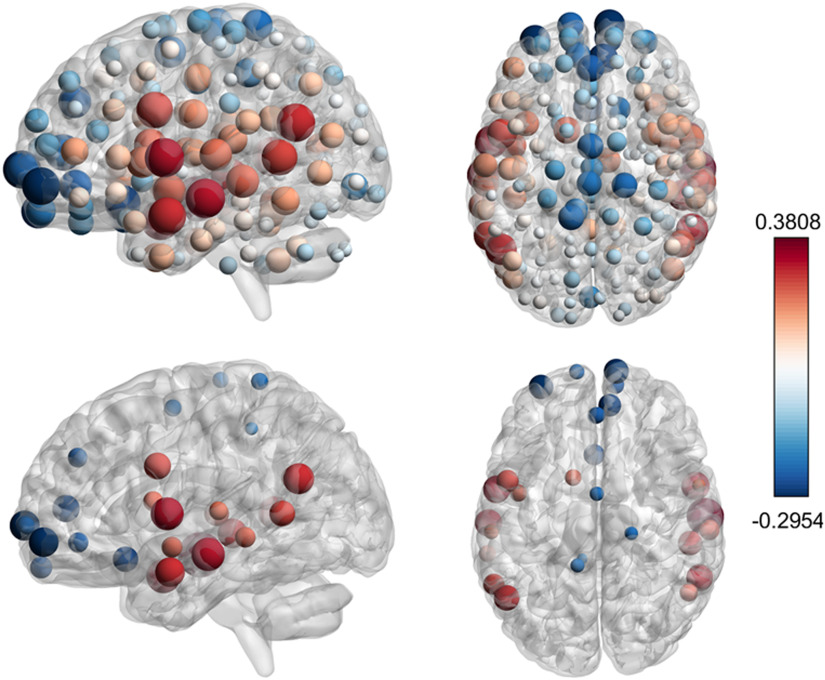
Correlations between synergy and age. (Top) Spearman correlation coefficient between the chronological age of the patient and the incoming synergy for each node, that is, the average of synergies for all triplets having that node as a target. Both positive (red) and negative (blue) correlations are found. The radius of the spheres is proportional to the absolute value of the correlation. (Bottom) Only the regions with significant correlation are shown, evaluated using Hochberg adjusted *p* values at a significance level *α* = 0.05. Synergy is positively correlated with age in the following regions: left middle temporal posterior, right middle temporal anterior, left angular, right superior temporal posterior, left middle temporal temporooccipital, right middle temporal posterior, left middle temporal temporooccipital, left middle temporal anterior, left central opercular, right superior temporal posterior; and negatively correlated in left frontal pole, left subcallosal, right frontal pole, paracingulate sulcus, juxtapositional lobule.

The implementation of the Ising model on the individual networks of healthy subjects shows the robustness of the scenario described above. Indeed, qualitatively similar results have been found on individual networks, each network showing, however, its own critical temperature as evaluated at the peak of the susceptibility. Remarkably, this analysis provides an individual pattern of synergy, evaluated at criticality, so as to evidence those nodes whose synergy covaries significantly with age. In Figure 7 we depict the Spearman correlation coefficient between incoming synergy and age, for each brain node, showing a symmetric pattern. We choose measures of association that are robust to the presence of outliers and suitable for multiple comparisons. We use the implementations contained in the robust statistical toolbox by Rand Wilcox (Rallfun-v37.txt, update September 2019), using R version 3.5.3 (R Core Team, 2018). In particular we adopt the skipped Spearman correlation with adjusted *p* values in conjunction with Hochberg’s method to control family-wise error. This approach is referred to as L3 in Wilcox, Rousselet, and Pernet (2018), and implemented in the R function scorregciH. This analysis yields 29 regions whose synergy is significantly associated with aging at a corrected *α* value of 0.05, seventeen regions positively correlated with age, and 12 negatively correlated with age, depicted in Figure 7.

The nodes whose synergy is significantly decreasing with age are frontal poles and subcallosal regions, as well as paracingulate sulcus and juxtapositional lobule (formerly supplementary motor area). It has been suggested that the frontal lobes are the part of the brain most profoundly affected by the aging process (Tisserand et al., 2002); a review of age-related changes in MR spectroscopy, functional mri, and diffusion tensor imaging can be found in Minati, Grisoli, and Bruzzone (2007). Successful cognitive aging and its functional imaging correlates are discussed in Eyler, Sherzai, Kaup, and Jeste (2011). Juxtapositional lobule is among the cortical motor regions whose dynamic has been shown to be modulated by age in Wang et al. (2019).

**Figure 8.**
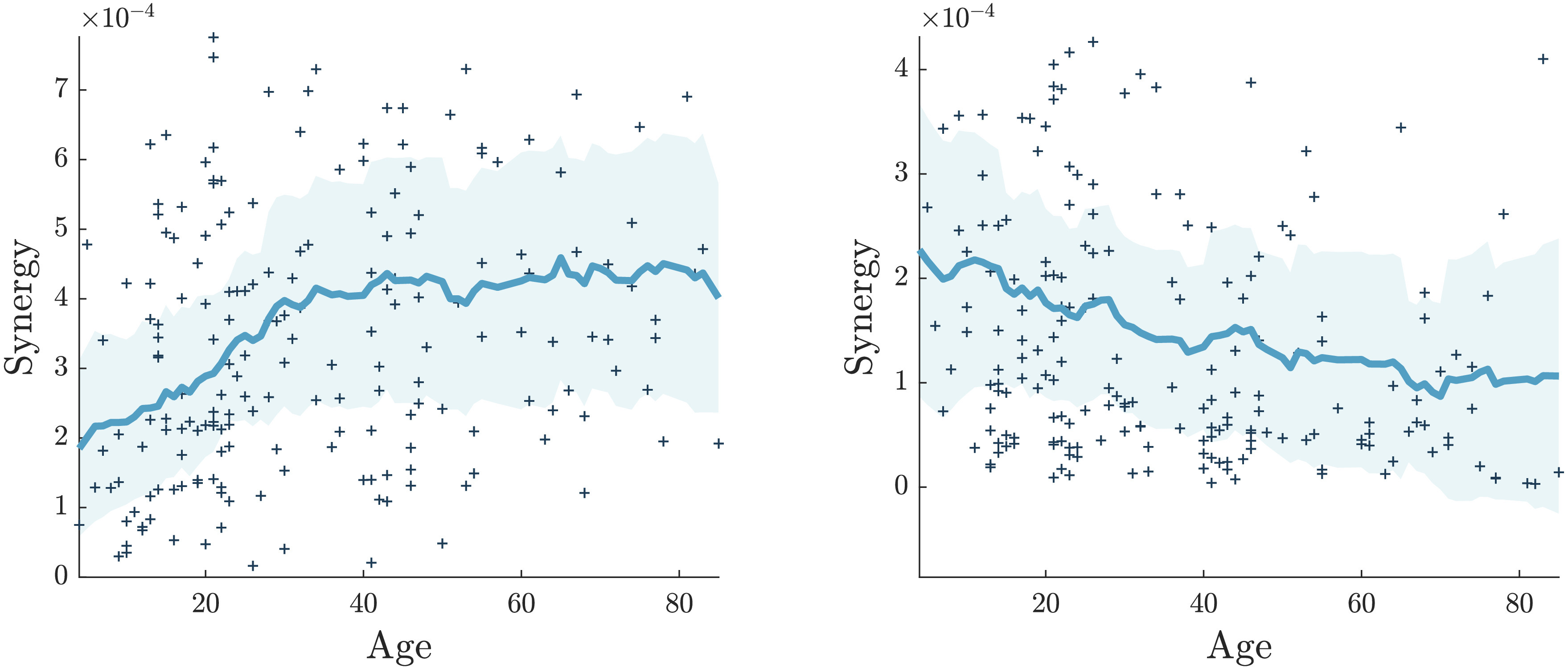
Scatterplot of synergy and age for two representative brain regions. left: Right superior temporal posterior, positive correlation. right: Right frontal pole, negative correlation. Local average and standard deviation are evaluated using the first 20 neighbors of each point.

The nodes whose synergy is significantly increasing with age are mostly located in the temporal cortex. In Figure 8 we have depicted, for example, the synergy versus age for two brain nodes, the right superior temporal posterior (showing a significant positive correlation with age) and the right frontal pole (showing a negative significant correlation with age). Figure 8 suggests that some regions show a slow and continuous decrease in synergy. On the other hand, the brain regions with a significant positive correlation actually show an increase in the synergy in the first decades of life, and a plateau later: Hence such an increase of synergy in the early age could be associated with brain maturation, while the plateau at late age can be seen as a compensatory effect due to the decline of white matter volume (Westlye et al., 2010) and of structural connectivity (Liu et al., 2017).

Finally, we looked at the correlation with age of the structural strength of the 29 regions whose synergy is correlated with age. Out of the 17 regions whose synergy significantly increases with age, 16 of them also show a significantly positive correlation between their strength and age; out of the 12 regions whose synergy significantly decreases with age, none of them displays a structural strength significantly correlated with age. This indicates that while the growth of the synergy in the positively age-correlated regions can be simply explained as a consequence of the increased connectivity of these regions, this does not hold for the synergy of the regions negatively correlated with age, where the relationship cannot simply be mapped on terms of some topological metrics and could arise in a more complex way from the underlying structural network.

## DISCUSSION

The analysis of computational models implemented on the realistic brain geometry is nowadays considered important to enrich our understanding of the mechanisms contributing to the formation and dissolution of functional brain networks. Some studies aimed at assessing to what extent whole-brain computational models reproduce graph-based topological features of the empirical functional resting-state networks; for example, see Lee, Bullmore, and Frangou (2017). In Meier et al. (2017), a susceptible-infected-susceptible (SIS) epidemic-spreading model on the human connectome has been studied to reveal the topological properties of the underlying structural network that gives rise to the global pattern of the directional information flow observed in data.

The Ising model represents the paradigmatic example of phase transitions; here we have implemented it on the large-scale structural geometry of the brain as measured by difussion tensor imaging, for individual networks of healthy subjects with different age, as well as on the average connectome, in order to analyze the simulated neural dynamics in terms of the synergy, a quantity that has recently been introduced in the field of partial information decomposition of information and that, in the case of the brain, measures to what extent the joint state of pairs of brain regions is projected onto the dynamics of a target region.

We find, as the temperature is lowered, that the synergy can still be considered as a precursor of transition in an inhomogeneous network and not only in an ordered lattice: The synergy towards the most connected brain regions (averaged over pairs of drivers) peaks before the maximum of the susceptibility, which is conventionally taken as the transition point in finite-size systems. The transition to order here is orchestrated by the hubs of the structural connectivity, and we have shown that the synergy of these hubs peaks at higher temperature than the critical temperature: Therefore the main findings of Marinazzo et al. (2019) can be generalized to heterogeneous networks provided that the synergy of structural hubs is considered. It is remarkable that the hubs of the synergy are not hubs of the strength; instead they are central nodes of the network, although neither the betweenness nor the closeness are fully monotonical with the synergy.

We also find that there are brain regions whose incoming synergy decreases continuously with age, in particular the frontal pole, the subcallosal area, and the supplementary motor areas: These brain nodes are known to be affected by the aging process (Kievit et al., 2014). Other brain regions, in the temporal cortex, show a positive correlation with age; however, looking at the scatterplot suggests that the increase stabilizes around the age of 30. As a consequence these results suggest that the temporal cortex experiences a remarkable maturation in term of the synergy, that is, on the capability of performing higher order computation. Most results about brain maturation refer to subcortical regions and prefrontal regions: The analysis of synergy here seems to suggest a role of the temporal cortex. The pattern of the synergy of the temporal cortex, described here, seems to correspond in the early age to the increase of the structural connectivity found in Liu et al. (2017) for the temporal lobe–related network. On the other hand, the same quantity showed a decline tendency in the late stage of life (Liu et al., 2017); the same decline was observed for the white matter volume in the temporal cortex in Westlye et al. (2010). A possible interpretation would be that the increase of synergy is compensatory for the decline of structural properties of the temporal cortex. Intuitively, synergy is the information about the target variable that is uniquely obtained by taking the sources together, but not considering them alone; hence it measures to what extent the activity of a given target region of the brain is the projection of the joint activity of two other driver regions, in other words the capability to perform computation of higher level. In this sense, these results suggest that the function of some regions of the brain may deteriorate with age with a contribution from the changing white matter geometry, while other regions receive more synergistic information as age increases.

We expect that a similar scenario for the synergy could hold also for models of phases and other dynamical systems on the connectome. More importantly, most studies so far have studied aging in terms of statistical dependencies (mainly correlations) between pairs of regions. Here we focused on higher order effects, which already become evident considering only the structural geometry of the brain: These higher order effects might be worth investigating also in empirical brain networks.

## DATA AND CODE AVAILABILITY

The processed connectomes are retrieved from http://umcd.humanconnectomeproject.org (NKI-Rockland; Brown & Van Horn, 2016). The code to simulate the dynamics of the Ising spins coupled according to the connectomes, as well as the synergy values for each region and each subject, is available at https://github.com/danielemarinazzo/ising_synergy_brain (Marinazzo, 2020; Nooner et al., 2012).

## AUTHOR CONTRIBUTIONS

Davide Nuzzi: Formal analysis; Software; Visualization; Writing - Original Draft; Writing - Review & Editing. Mario Pellicoro: Formal analysis; Software. Leonardo Angelini: Conceptualization; Methodology; Project administration. Daniele Marinazzo: Conceptualization; Formal analysis; Investigation; Methodology; Software; Visualization; Writing - Original Draft; Writing - Review & Editing. Sebastiano Stramaglia: Conceptualization; Investigation; Methodology; Supervision; Validation; Writing - Original Draft; Writing - Review & Editing.

## FUNDING INFORMATION

Sebastiano Stramaglia, Ministero dell’Istruzione, dell’Universit e della Ricerca (http://dx.doi.org/10.13039/501100003407), Award ID: PRIN 2017/WZFTZP.

## TECHNICAL TERMS

Ising model: A mathematical model of ferromagnetism, consisting of discrete variables called spins that can be in one of two states (+1 *or* −1) and that are arranged in a graph allowing each spin to interact with its neighbors. The two-dimensional square-lattice Ising model is one of the simplest statistical models to show a phase transition.
Critical brain hypothesis: Evidence has been mounting that biological systems, in particular neuronal networks, might operate at the borderline between order and disorder, that is, near a critical point.
Brain connectivity network (connectome): A network in which the nodes are brain regions and the links are anatomical connections (“anatomical/structural connectivity”) or statistical dependencies (“functional connectivity”).
Synergy: A quantity rooted in information theory measuring how much the joint state of pairs of brain regions is projected onto the dynamics of a target region.
Transfer entropy: A nonparametric statistic measuring the amount of directed (time-asymmetric) transfer of information between two random processes.
The law of diminishing returns: A fundamental principle of economics stating that in all productive processes, adding more of one factor of production, while holding all others constant (”ceteris paribus”), will at some point yield lower incremental per unit returns. A similar scenario is observed in the information flow pattern of dynamical networks.
Partial information decomposition: A recent extension of Shannon information theory quantifying the information that several inputs provide individually (unique information), redundantly (shared information), or only jointly (synergistic information) about the output.
